# Application of a novel biological-nanoparticle pretreatment to *Oscillatoria acuminata* biomass and coculture dark fermentation for improving hydrogen production

**DOI:** 10.1186/s12934-023-02036-y

**Published:** 2023-02-22

**Authors:** Mostafa El-Sheekh, Mostafa Elshobary, Eman Abdullah, Refat Abdel-Basset, Metwally Metwally

**Affiliations:** 1 Department of Botany, Faculty of Science, Tanta University, Tanta, 31527 Egypt; 2 Botany and Microbiology Department, Faculty of Science, Assuit University, Assuit, Egypt

**Keywords:** Biohydrogen, Biological pretreatment, Thermochemical, Coculture, Dark fermentation, Nanoparticles

## Abstract

**Background:**

Energy is the basis and assurance for a world's stable development; however, as traditional non-renewable energy sources deplete, the development and study of renewable clean energy have emerged. Using microalgae as a carbon source for anaerobic bacteria to generate biohydrogen is a clean energy generation system that both local and global peers see as promising.

**Results:**

*Klebsiella pneumonia, Enterobacter cloacae*, and their coculture were used to synthesize biohydrogen using *Oscillatoria acuminata* biomass via dark fermentation. The total carbohydrate content in *O. acuminata* was 237.39 mg/L. To enhance the content of fermentable reducing sugars, thermochemical, biological, and biological with magnesium zinc ferrite nanoparticles (Mg-Zn Fe_2_O_4_-NPs) pretreatments were applied. Crude hydrolytic enzymes extracted from *Trichoderma harzianum* of biological pretreatment were enhanced by Mg-Zn Fe_2_O_4_-NPs and significantly increased reducing sugars (230.48 mg/g) four times than thermochemical pretreatment (45.34 mg/g). *K. pneumonia* demonstrated a greater accumulated hydrogen level (1022 mLH_2_/L) than* E. cloacae* (813 mLH_2_/L), while their coculture showed superior results (1520 mLH_2_/L) and shortened the production time to 48 h instead of 72 h in single culture pretreatments. Biological pretreatment + Mg-Zn Fe_2_O_4_ NPs using coculture significantly stimulated hydrogen yield (3254 mLH_2_/L), hydrogen efficiency)216.9 mL H_2_/g reducing sugar( and hydrogen production rate (67.7 mL/L/h) to the maximum among all pretreatments.

**Conclusion:**

These results confirm the effectiveness of biological treatments + Mg-Zn Fe_2_O_4_-NPs and coculture dark fermentation in upregulating biohydrogen production.

**Supplementary Information:**

The online version contains supplementary material available at 10.1186/s12934-023-02036-y.

## Background

Recent years have seen a rise in the use of sustainable and clean fuels, particularly biofuels, due to the high expense of fossil fuels and their detrimental impact on the environment [^1^, ^2^]. Scientists are becoming more interested in biofuels because they are environmentally friendly and made from affordable, non-edible, and inexpensive feedstocks [3–5]. Hydrogen is typically deemed as a cleaner and more promising contender due to the most incredible energy output of any other fuel (143 GJ tonne^−1^). In addition, it is the only known fuel that doesn't emit CO_2_ as a byproduct in combusting engine. Biological hydrogen synthesis from algae is interpreted as one of the most talented green power generation solutions [6]. Hydrogen synthesis from algae is investigated as an alternative form of energy for power generation with the development and commercialization of fuel cells. Presently, sugarcane and maize as starch-rich crops and lignocellulosic raw materials (such as rice straw and agriculture wastes) are utilized as biomass feedstocks for biofuel.

Nevertheless, exploiting agricultural crops or wastes for biofuel production presents challenges owing to limited arable land and water supplies [7]. Furthermore, the increased cost of digestion of lignocellulosic biomass is one of the technique's weaknesses owing to the high lignin concentration in biomass, making the saccharification process challenging [8, 9]. Lately, microalgae, including cyanobacteria, have been proposed as a third-generation feedstock for biofuel production [6, 8]. Furthermore, microalgae species accumulate significant content of carbohydrates in the form of starch, glycogen, and cellulose, that consider efficient biohydrogen substrates [9]. Lacking lignin is another advantage of using microalgae/cyanobacteria biomass, making it simpler to convert to monosaccharides for fermentation [10]. Cyanobacteria have primarily been studied for their ability to convert solar energy into fixed carbohydrates. Compared to higher plants, cyanobacteria have the higher photosynthetic capacity and faster cell growth rates. Therefore, they can be easily cultivated using only the most essential nutrients, air, water, and light [11].

*Oscillatoria* sp. is a filamentous cyanobacterium that has been investigated for its potential use in biofuel production [12]. Studies have shown that it has a high carbohydrate level, which can be utilized in biohydrogen synthesis through the process of dark fermentation [6]. Additionally, *Oscillatoria* sp. has been found to be a suitable strain for biohydrogen production due to its high rate of growth and capability to survive in a wide-ranging of environments [13]. Carbohydrates in cyanobacteria are mainly derived from glycogen accumulated in the cytoplasm and various cell wall polysaccharides, which are not easily fermentable by microbes for biohydrogen generation [9]. As a result, various pretreatment methods are being used to break and hydrolyze the intricate cell wall carbohydrates in order to produce simple fermentable sugars, consist of physical (grinding, sonication and pyrolysis), chemical (alkali, acid and thermal), and biological (enzymes) pretreatments [14]. However, the most effective approach for cyanobacteria has yet to be determined. As a result, several pretreatment protocols have been used with variable degrees of achievement. Pretreatment optimization can help with cost-effective hydrogen production since its eco-friendly, power-saving, and has zero carbon emission. The conversion of cyanobacterial biomass into sustainable biohydrogen by H_2_-producing bacteria through dark anaerobic fermentation is getting prominence [15] due to its energy-efficient, environmentally benign, and carbon–neutral properties. It is a talented energy source candidate to substitute traditional fossil fuels, which is advantageous from both an economic and environmental perspective [16]. Most biological H_2_ generation activities occur at ambient temperatures and pressure [17]. These milder conditions are better for the environment and save energy.

Recently, scientists have been exploring the use of nanomaterial catalysts as a way to enhance the conversion of cellulosic and lignocellulosic biomass into sugar [18–20]. Nanoparticles have unique physical, chemical, and electrical properties compared to their bulk counterparts. They can be used as catalysts to catalysts in the fermentation process and have a positive effect on the stability and efficiency of the microorganisms involved in the fermentation process [21–23]. Various types of nanomaterials, such as magnetic, carbon nanotubes, and metal oxide nanoparticles, have been studied for their potential in sustainable bioenergy production [24]. However, few studies have been conducted on the use of nanoparticles to enhance the enzymatic conversion of algal biomass for sugar production in fermentation processes [18].

On the other hand, identifying and isolating hydrogen-producing bacteria is another challenging step in biohydrogen production. Wastewater sludge is one of the rich sources of hydrogen-producing bacteria. This includes isolates from various genera, such as *Clostridium*, *Bacillus*, *Enterobacter* and *Klebsiella*, among others [25]. The enterobacterium species such as *Enterobacter cloacae* and *Klebsiella pneumoniae* were selected from environmental sources and has shown potential for producing biohydrogen from different substrates [25–27].

On the basis above facts, this work aimed to utilize the *Oscillatoria acuminata* biomass as substrate in hydrogen production. Biological pretreatment + Mg-Zn Fe_2_O_4_ NPs was applied to* Oscillatoria acuminata* biomass for the first time, according to our knowledge, to enhance their soluble and fermentable sugars for rapid bacterial consumption in hydrogen production. The results were compared with thermochemical and biological pretreatments. Biohydrogen production from* Klebsiella pneumonia, Enterobacter cloacae* individually or their coculture were studied to trigger the conversion of cyanobacterial biomass into biohydrogen. Furthermore, the biohydrogen evolution efficiency was computed in terms of dry mass and reducing sugars.

## Results and discussion

### Isolation and identification of microalgal species

**Fig. 1:**
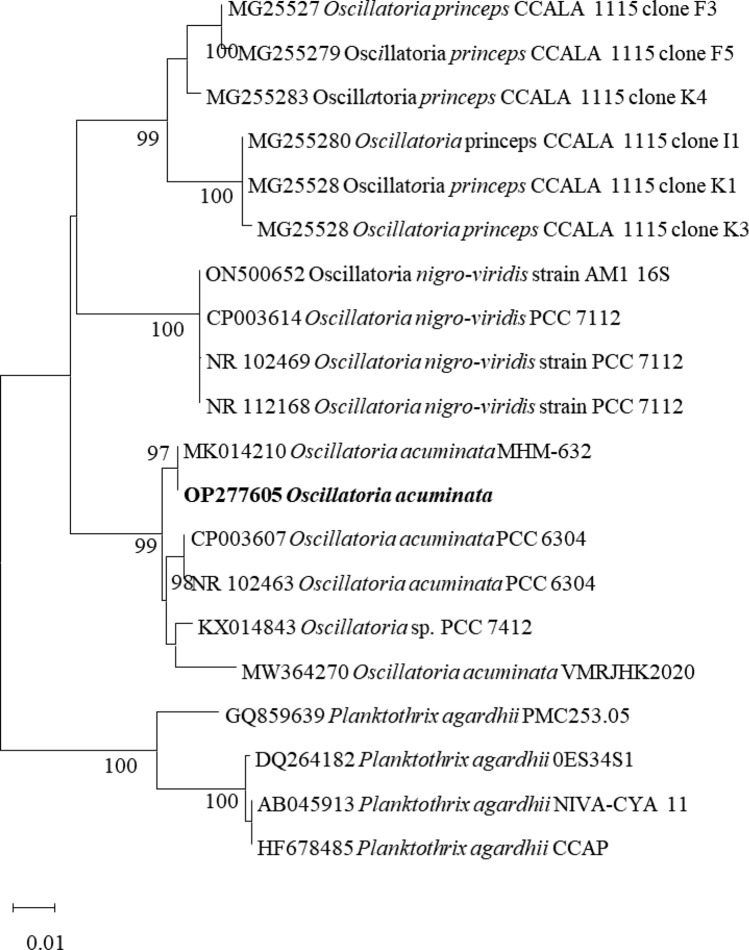
Neighbor-Joining (NJ) dendrograms showing the isolated *Oscillatoria acuminata* based on 16S rRNA nucleotide sequences, respectively. Bootstrap values higher than 70 are shown below the branches of the trees

*Oscillatoria* was genetically identified using 16S rRNA gene, respectively. The sequence analysis was done with the neighbor-joining algorithm based on the parameter distance (NJ-PD) by aligning the 16S gene sequence with 16S nucleotide sequences of 16 cyanobacteria species plus four 16S sequences of *Planktothrix agardhii* as an outgroup (Fig. 1). Each *Oscillatoria* sp. formed monophyletic subclades with bootstrap support, ranging from 87 to 100. The sequences were subjected to BLAST homology searches of the 16S sequence, indicating that the closest match was *Oscillatoria acuminata* (Fig. 1). *Oscillatoria* sp showed very high similarity (~ 98–100%) to *Oscillatoria acuminata* (MK014210, NR_102463, and CP003607). The sequences of the cyanobacteria have been submitted to Genbank (OP277605).

### Estimation of growth and biochemical composition of *Oscillatoria acuminata*

**Fig. 2:**
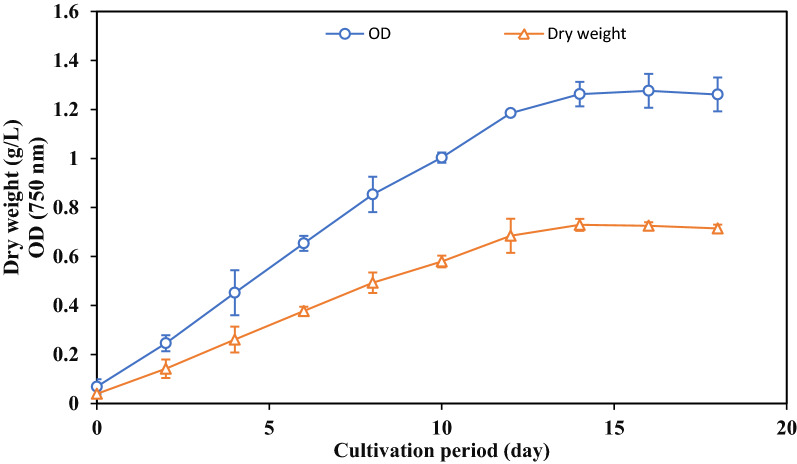
Growth curve of *O. acuminata* using dry weight and optical density (OD 750)

The algal growth was determined by optical density and dry weight. The results in Fig. 2 indicated that the growth curve of *O. acuminata* showed no lag phase. The exponential phase was from zero to the 14th day. These cultivation periods are shorter than those recorded by El-Sheekh et al. [28], who denoted that *O. acuminata* could grow till the 22nd day of cultivation. This difference in biomass may be due to the differences in culture medium, cultivation condition, and metabolic activity [29, 30].

The biochemical composition was estimated at the end of the log growth phase on the 16th day of culture (Table 1). Chl a and carotenoid content extracted from *O. acuminata* recorded maximum value at 16th (18.1 µg/mL, 3.3 µg/mL respectively). *O. acuminata* showed the highest carbohydrate content of 237.39 mg/L, while the total protein content was 180.78 mg/L. These results are less or more than those recorded in recent studies [29]. This could be attributed to the difference between the isolates, biomass yield, and growth conditions [31]. Microalgal biomass was harvested on the 16th day for use in further experiments.

**Table 1:** Metabolic constituents of *O. acuminata*

Biochemical constituents	Contents
Chlorophyll a (µg/mL)	18.1 ± 0.3
Carotenoid (µg/mL)	3.3 ± 0.07
Total soluble protein (mg/L)	180.78 ± 2.21
Total soluble carbohydrate (mg/L)	237.39 ± 4.12

Values represent means of three replicates ± standard error

### Influence of different pretreatment methods on biomass hydrolysis

Pretreatment is crucial in releasing fermentable sugars to be used in microbial fermentation.

#### Thermochemical pretreatment of microalgal biomass

**Fig. 3:**
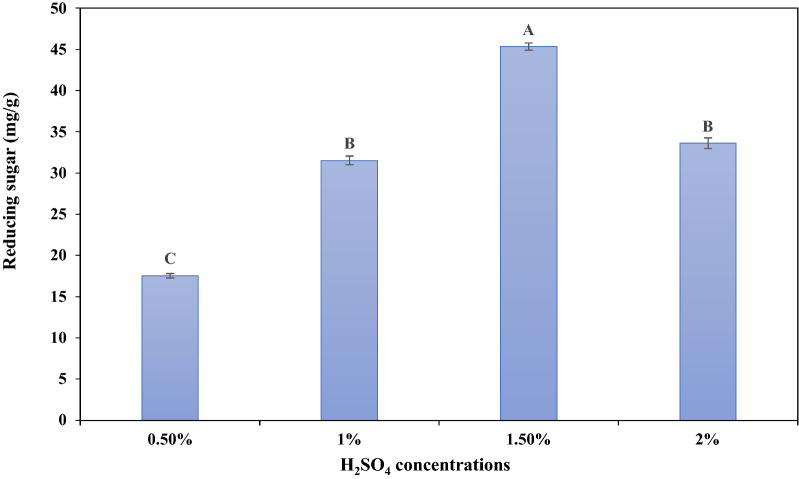
Effect of different concentrations of acid pretreatments on *O. acuminata* biomass for reducing sugar production. Different capital letters of the plotted series indicate significant differences at p ≤ 0.05 using Duncan's test

As shown in Fig. 3, the highest value of reducing sugar was recorded at 1.5% H_2_SO_4_ of 45.34 mg/g dry weight DW, while any increase or decrease in H_2_SO_4_ concentrations reduces sugar content reduced. Among various acids hydrolysis, Hessami et al. [32] proved that H_2_SO_4_ is the best hydrolysis acid compared to HCl, HClO_4_ and CH_3_COOH. Thus, acid hydrolysis using sulphuric was performed in the current study as an efficient, fast, appropriate and cost-effective technique [33]. Furthermore, El-Souod et al. [34] demonstrated that hydrolysis of microalgal biomass using H_2_SO_4_ pretreatment showed the best results, especially at 1.5%. These results run with Ashour et al. [35], Elshobary et al. [33] and Li et al. [36], who found that dilute acid pretreatment was extensively used since strong acid would induce the excessive breakdown of the complex material, resulting in a release of fermentable sugars. However, acid pretreatment produces fermentation-inhibiting compounds for example, furfural, hydroxymethylfurfural, and levulinic acid [37]. In contrast, biological pretreatment, such as fungal treatment, may be a further practical alternative than typical acid pretreatment processes [38].

#### Biological treatment of microalgal biomass

This study studied biological pretreatment as an eco-friendly tool to maximize reducing sugar release, avoid generating fermentation-inhibiting compounds and reduce chemical inputs. Fungi produce several hydrolysis enzymes such as cellulase, filter paper cellulase and β-glucosidase. These enzymes break down the algal cell wall and convert complex polysaccharides to fermentable monosaccharides. *T. harzianum* (OP264067) showed the highest cellulolytic activity compared to 20 isolated fungi [39] and naturally produces hydrolysis enzymes such as cellulase, β-glucosidase and xylanase [^40^].

#### Screening of different substrates for cellulolytic activity produced by *T. harzianum* using solid-state fermentation (SSF)

**Fig. 4:**
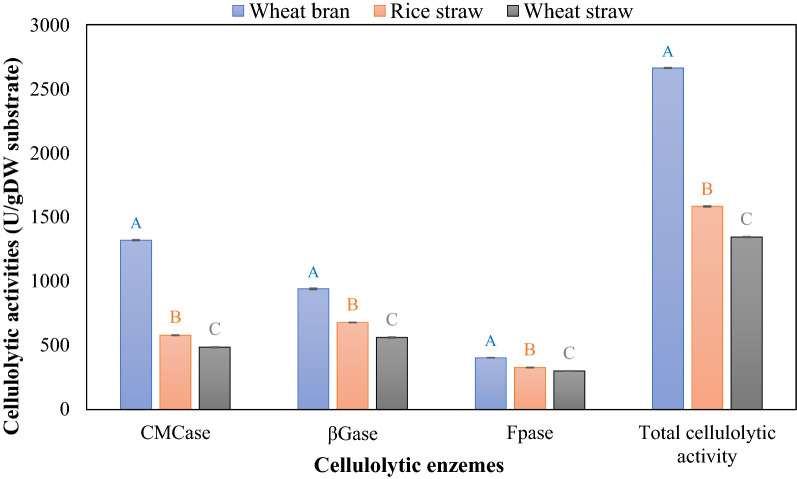
Screening of different substrates for cellulolytic activity produced by *T. harzianum* using solid-state fermentation (SSF). Different capital letters of the plotted series indicate significant differences at p ≤ 0.05 using Duncan's test

To further increase the hydrolysis process and reducing sugar content, optimization of biological pretreatment was conducted using different substrates (rice straw, wheat straw, and wheat bran) Fig. 4. Results demonstrated that wheat bran was the most efficient substrate that improved the cellulolytic activity of *T. harzianum* to the maximum. The maximum activities of CMCase, βGase, Fpase were 1320.57, 940.59 and 402.41 U/g DW substrate, respectively and total cellulolytic activity was 2663.56 U/g DW using wheat bran, followed by rice straw (1582.52 U/g DW), and wheat straw (1345.06 U/g dry weight substrate). Our results agreed with El-Shishtawy et al. [41], who reported that wheat bran substrate inhanced the hydrolysis enzymes, total proteins, and carbohydrates of *T. virens*to the maximum using base pretreated. Moreover, wheat bran revealed its potential as a cheap substrate for more remarkable enzyme synthesis by *Penicillium citrinum* [42]. One unit of FPase, βGase, and CMCase was described as µg of reducing sugars produced per minute per gram of substrate dry weight.

#### Effect of Mg-Zn Fe_2_O_4_-NPs on the hydrolytic enzymes of *T. harzianum*

**Fig. 5:**
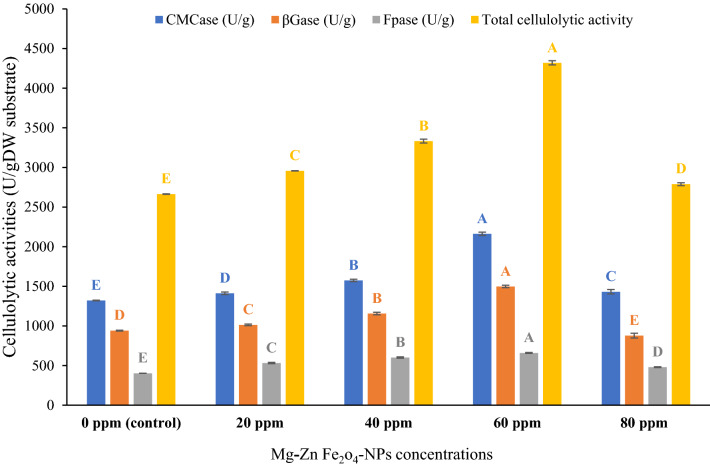
Effect of Mg-Zn Fe_2_O_4-_NPs for cellulolytic activity produced by *T. harzianum* Ps-2 using solid-state fermentation (SSF). All data represented means of 3 replicas ± standard deviation (SD). Different capital letters in each plotted series indicate significant differences at p ≤ 0.05 using Duncan's test

Cellulolytic action of *T. harzianum* using wheat bran as substrate was further improved by Mg-Zn Fe_2_O_4_-NPs as shown in Fig. 5. The results concluded a gradual increase in all cellulolytic activities (CMCase, βGase, Fpase and total cellulolytic activity) by applying different Mg-Zn Fe_2_O_4_-NPs concentrations until reaching the maximum activities at 60 ppm of Mg-Zn Fe_2_O_4_-NPs for CMCase, βGase, Fpase (2162.40, 877.65 and 659 U/g respectively) with the highest total cellulolytic activity (4319.37 U/g). Interestingly, 60 ppm of Mg-Zn Fe_2_O_4_-NPs improved the total cellulolytic activity about two times over the untreated control. It has been observed that the presence of metal oxides is essential for the production of cellulase enzymes and growth of microorganisms in fermentative media. This is due to the fact that these micronutrients play a crucial role in the synthesis of cellulase enzymes and microbial growth. Therefore, by incorporating Mg-Zn Fe_2_O_4_-NPs as a catalyst, it enhanced the production of cellulase enzymes and biohydrogen [20]. Moreover, the stability of enzymes can be significantly improved through protein adsorption on nanomaterials, due to the unique properties provided by high surface area to volume ratios [43]. Furthermore, the high surface area of nanoparticles offers an efficient matrix for immobilizing enzymes, resulting in enhanced stability. The large surface area of these materials allows for multiple points of attachment for enzyme particles, which prevents protein unfolding and ultimately leads to improved enzyme stability [44]. Srivastava et al. recorded that nickel ferrite nanoparticles stimulated the yield of total cellulase enzyme using remaining cyanobacteria biomass of *Lyngbya limnetica* as feedstock [18]. Asar et al*.* reported a comparable increase in cellulase and sugar yield following the application of iron oxide magnetic nanocomposites [45].

#### Effect of enzyme dose and incubation duration on cyanobacterial biomass

**Fig. 6:**
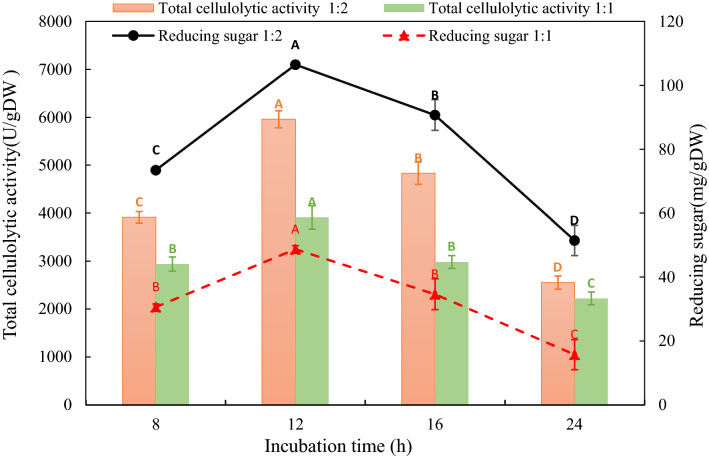
Effect of enzyme dosage and incubation time on biodegradation of *O. acuminata* biomass*.* All data represented means of 3 replicas ± standard deviation (SD). Different capital letters in each plotted series indicate significant differences at p ≤ 0.05 using Duncan's test

One of the most common and straightforward experiments used to improve the fermentation process is single-factor optimization [46, 47]. Effect of enzyme dose and incubation duration were determined and optimized by a series of single-factor experiments to provide optimal conditions for the fermentation process. The results illustrated in Fig. 6 showed that the highest yield (106.48 mg/g) of reducing sugar and cellulolytic activity (5958.50 U/g) were recorded at 1:2 crude enzyme: algal suspension (33%) concentration after 12 h. Cellulolytic activity at an enzyme concentration of 33% increased by 52.31%. These results may be explained by increasing algal suspension increases the substrate and available carbon source for the enzyme activity. In this context, Vishwakarma and Malik investigated the enzymatic pretreatment effects using crude enzymes from *T. reesei* increased by twofold in protein efflux and a 41% increase in released sugars after 12 h of enzymatic pretreatment at 33% (v/v) enzyme concentration [48].

#### Cellulolytic activity and reducing sugar in the presence of Mg-Zn Fe_2_O_4_-NPs by solid state fermentation

Optimized conditions of 1:2 crude enzyme: algal suspension at 12 h incubation were subjected to Mg-Zn Fe_2_O_4_-NPs to maximize the cellulolytic activity and reducing sugar content of *O. acuminata* biomass. As a result, Mg-Zn Fe_2_O_4_-NPs significantly induced the production of reducing sugar and cellulolytic activity by 20.1% (7158.50 U/g) and 1.16-fold of reducing sugar (230.48 mg/g, 23.05 g/L) compared to biological pretreatment without NPs.

Fewer studies have been conducted on the use of nanoparticles to treat enzymatic conversion of biomass for sugar production, but the results of this study showed a higher production of sugar and higher cellulolytic activity compared to previous research. For example, Srivastava et al. recorded enhanced cellulolytic activity to 222 U/g by using nickel ferrite nanoparticles using residual biomass of *Lyngbya limnetica* [18]. Zanuso et al. obtained 21.84 g/L reducing sugar production using corn cob biomass using magnetic nanoparticles [49], and Jiang et al. showed an improvement in sugar production using corn stalk with the use of cellulase enzyme immobilized on nanocomposite compared to using the crude enzyme [50]. It is important to note that the type and form of magnetic carboxymethyl chitosan/calcium alginate–cellulase nanomaterials used can greatly impact the efficiency of hydrolysis enzymes, along with the type of fungal strain used. As previously discussed, the type and form of nanomaterials have a significant impact on the effectiveness of enzymes, in addition to fungal species used.

#### Fourier Transform Infrared Spectroscopy (FTIR) of the algal biomass before and after pretreatment

**Fig. 7:**
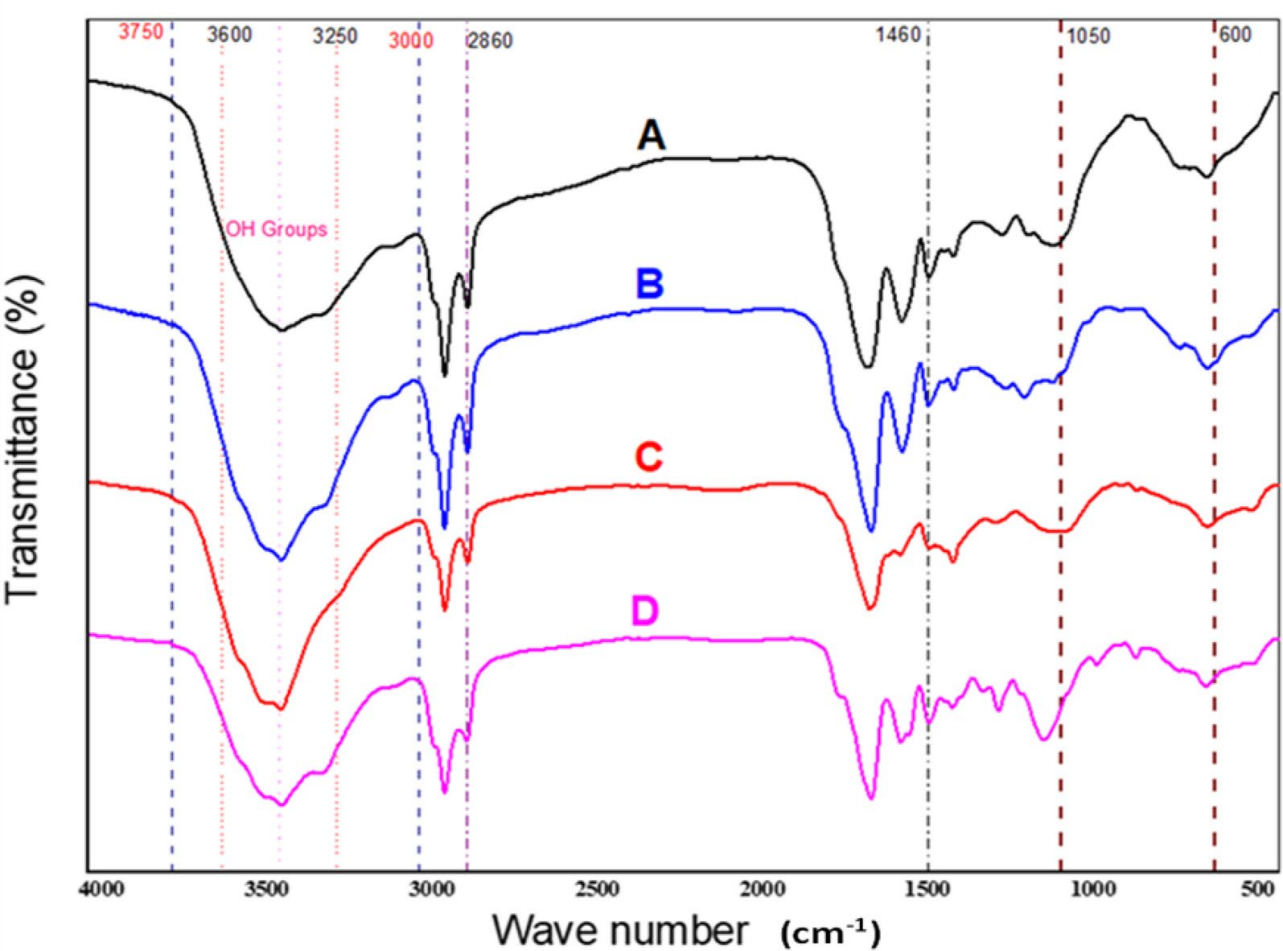
The diagnostic fingerprint regions of the FT-IR spectra of raw and pretreated *O. acuminata* biomass. A, untreated biomass; B, Chemical pretreatment; C, Biological pretreatment, and D, Biological pretreatment enhanced by Mg-Zn Fe_2_o_4_-NPs

FTIR was used to demonstrate the structural and chemical variations in different pretreatments (chemical, biological, and biological pretreatment + Mg-Zn Fe_2_O_4_-NPs) using *O. Acuminata* biomass. The Hydrogen bond band at 3600–3250 cm^−1^ was diminished after the hydrolysis due to the destruction of many H-bonds in the cellulose molecules [51]. Substantial reduction in the intensity at 3600–3250 cm^−1^ in biological + Mg-Zn Fe_2_O_4_-NPs pretreatment, indicating the breaking down of hemicellulose by cracking of H-bond in the amorphous cellulose [52, 53]. Variations in band intensity and position may imply a reduction in structural component concentration as well as the development of different kinds of groups from free radical groups created during treatments [54]. The expansion of the band also shows the bond's weakening as a result of pretreatment. Because these peaks reflect proteins, secondary amines (proteins, lipids), saccharides, and carbohydrates, a drop in intensity correlates to a reduction in their concentration. The peak at 2860 cm^−1^ was nearly gone after biological treatment, indicating that the carbohydrate and lipid complexes had been broken down [55]. The functional groups of methylene (-CH_2_-), methyl (-CH_3_), and glycosidic bond dominated the spectrum area at 1460–1160 cm^−1^. These groups are extensively dispersed and generated in numerous monosaccharides of the hemicellulose molecule, such as xylose, arabinose, and mannose. [53, 56]. The glycosidic link (C–O–C) breaking down in amorphous cellulose causes an obvious vibration at wavenumbers of 600–1050 cm^−1^. The intensities of the typical cellulose peaks were found to diminish after treatments (Allard and Templier, 2001). The shift in peak intensity at wavenumbers 700–600 cm^−1^ was related to the cis–trans structural alteration in the cell wall carbohydrates during biomass processing. The band at 600–900 cm-1 was ascribed to the vibration of ether groups or glycosidic bonds, which were the primary connections in polysaccharide compounds and pectin [57]. These results verified that Mg-Zn Fe_2_O_4_-NP_S_ enhanced the efficiency of biological pretreatment of *O. acuminata* biomass by sequentially cracking the cell wall structural components (Fig. 7).

#### Scanning Electron Microscopic (SEM) analysis of the cyanobacterial biomass before and after treatment

**Fig. 8:**
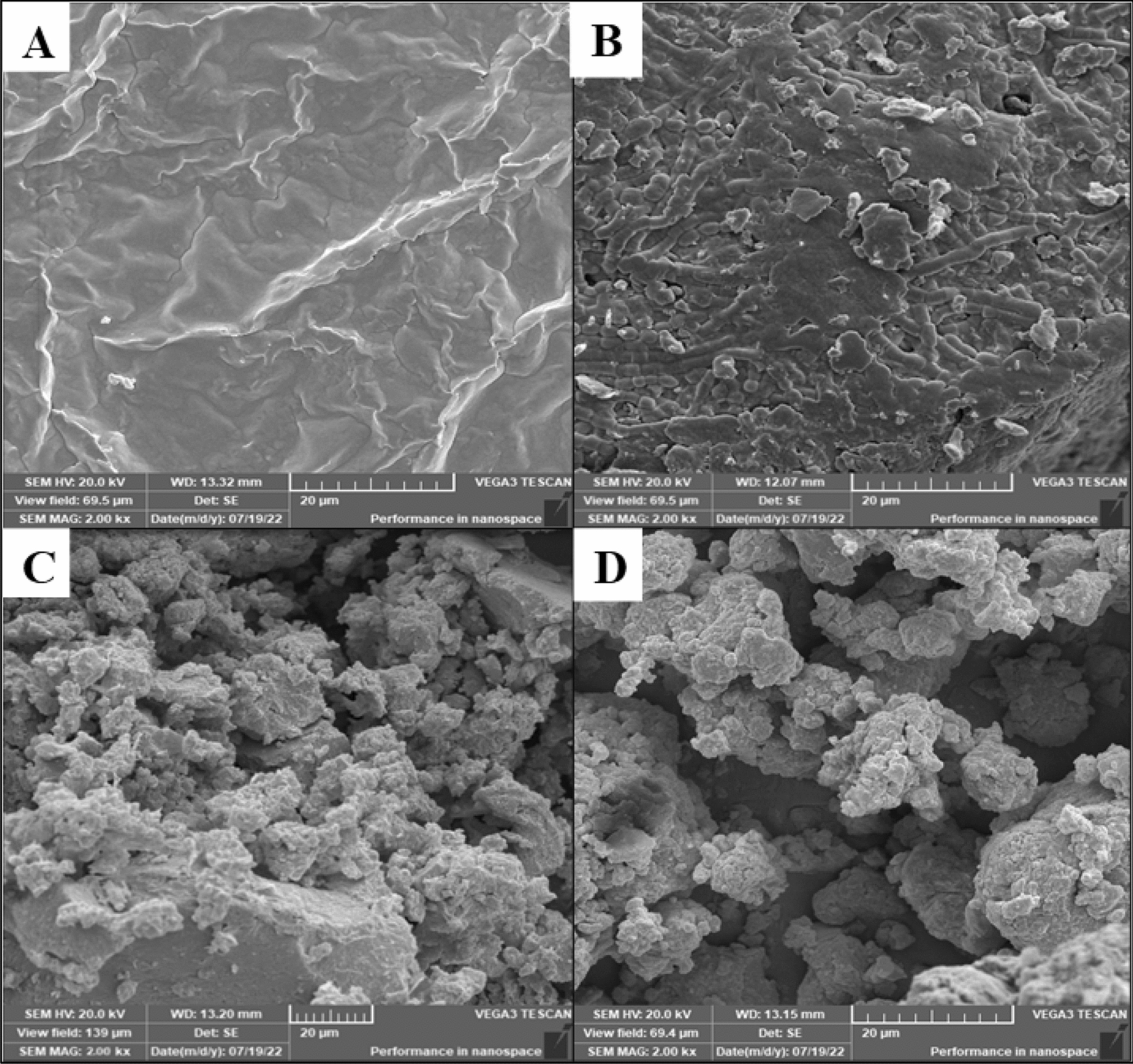
Scanning Electron Microscopy (SEM) observation of *O. acuminata* biomass at 2000 × magnification. Where, **A**, untreated biomass; **B**, Chemical pretreatment; **C**, Biological pretreatment and **D**, Biological pretreatment enhanced by Mg-Zn Fe_2_o_4_-NPs

In order to get insights into the structural and morphological variations in *O. acuminata* biomass during pretreatments (thermochemical, biological, and biological pretreatment + Mg-Zn Fe_2_O_4_-NPs). SEM microscopy analysis was used. Figure 8 reveals that the untreated biomass had a normal, smooth, and compact surface structure without degradation (Fig. 8A). After chemical treatment, the cell wall was partially broken (Fig. 8B). In biological treatment, obvious damage observed, resulting in exposure of the inner structure (Fig. 8C) [58]. The structure and integrity of *O. acuminata* cell walls might be efficiently disrupted with biological (fungal) pretreatment. The structure was utterly damaged after applying the biological pretreatment enhanced by Mg-Zn Fe_2_O_4_-NPs. This biomass treatment led to severe cell morphology alterations, including severe cell breaking and visible cellular debris (Fig. 8D) [59].

## Biohydrogen production

### Isolation and purification of biohydrogen-producing bacteria

**Fig. 9:**
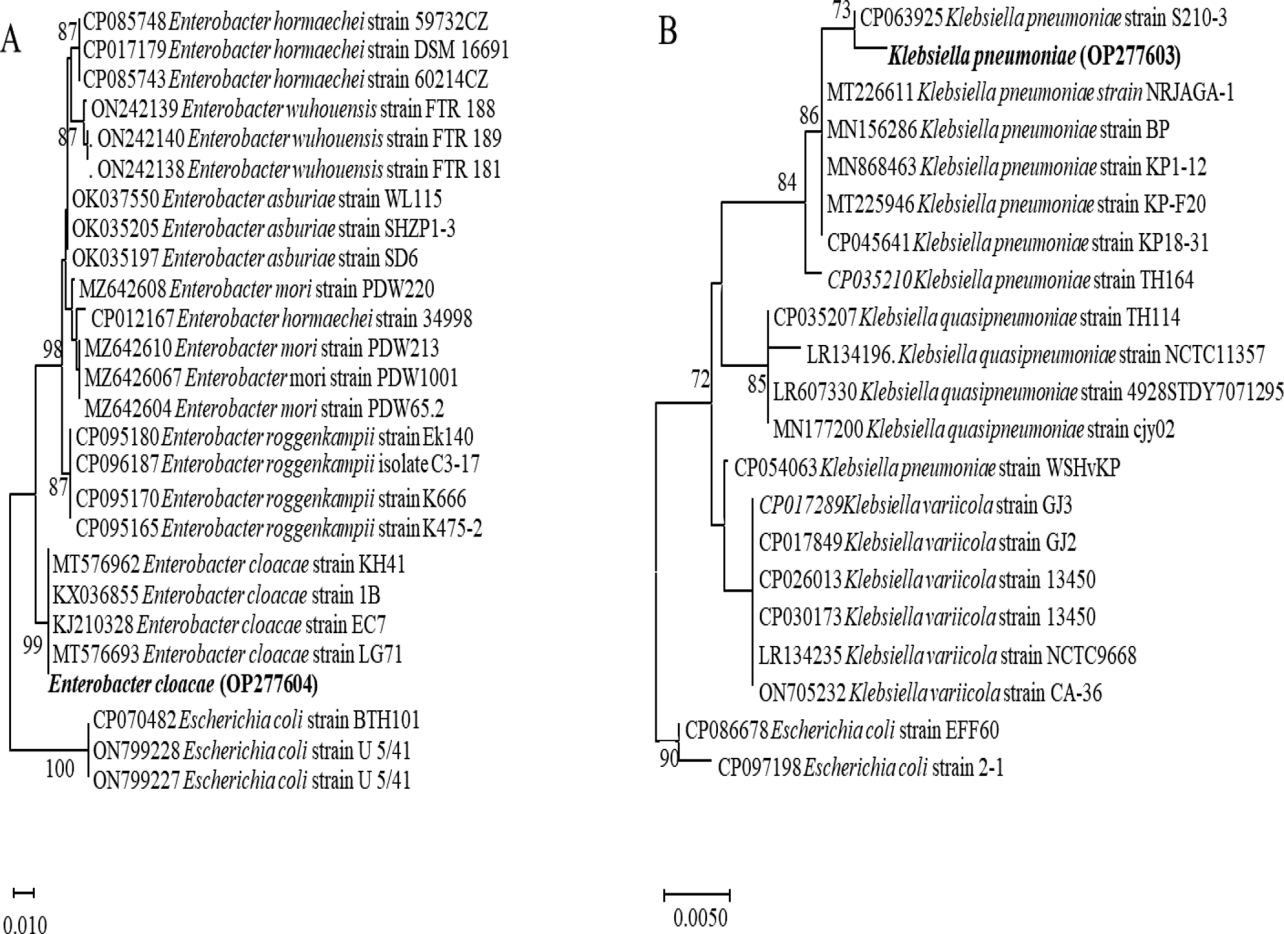
Neighbor-joining (NJ) phylogenetic tree for* E. cloacae* (**A**) and *K. pneumoniae* (**B**) based on 16S nucleotide sequences. Bootstrap values greater than 70 are shown. The identified species in this study were marked in bold

Two bacterial species were isolated from sewage sludge. Biochemical characterizations were performed using Bergey’s Bacteriology System to confirm that these isolates belonged to the Enterobacteriaceae family and were identified as* Klebsiella pneumonia* and* Enterobacter cloacae)*. In addition, a phylogenetic tree was built using the 16S rRNA gene sequence analysis to verify their taxonomic position. For the *Enterobacter cloacae*, the sequence analysis was accomplished with the neighbor-joining algorithm based on the parameter distance (NJ-PD) by aligning the 16S rRNA gene with 16S nucleotide sequences of 26 bacteria species plus five 16S rRNA sequences of *Escherichia coli* as an outgroup (Fig. 9A). These sequences showed identical similarity (100%) to *Enterobacter cloacae* (Genbank ON422258, KX036855, and KJ210328). Therefore, the isolated *Enterobacter cloacae* sequence was banked in the Genbank database under the accession number OP277604.

On the other hand, the *K. pneumoniae* sequences were subjected to BLAST homology searches of the 16S sequence, indicating that the closest match was *K. pneumoniae* (Fig. 9B). The phylogenetic tree comprises 20 sequences of *Klebsiella* sp. and two *Escherichia coli* 16S sequences as an outgroup. *Klebsiella* sp. sequences showed high similarity (~ 99%) to *Klebsiella pneumoniae* (Genbank CP063925, MT226611 and CP045641) that were grouped in the subclade of the first clade with 86 bootstraps. Therefore, the isolated *K. pneumoniae* sequence was banked in the Genbank database under the accession number OP277603.

### Biohydrogen production using hydrolysate of *O. acuminata* by different treatments

#### Chemical pretreatment

The cumulative biohydrogen production from *K. pneumoniae* and *E. cloacae* as well as their coculture showed significant differences by increasing incubation periods (p < 0.001), as illustrated in Table 2. Biohydrogen production from *K. pneumoniae* using thermochemical pretreatment hydrolysate of *O. acuminata* (1022 mLH_2_/L) was higher than* E. cloacae* (813 mLH_2_/L). The obtained results are in harmony with that detected by Ramu et al. [60], who demonstrated that *K. pneumoniae*-FA2 showed the maximum yield of biohydrogen (1094 mLH_2_/L) using food wastewater as a feedstock. The efficiency of *K. pneumoniae* was due to its greater ability to decompose dissolved sugars as a carbon source to generate biohydrogen. While the coculture of *K. pneumoniae* and *E. cloacae* showed superior hydrogen production (1520 mLH_2_/L) with an increase of 48% and 53% compared to the monoculture of *K. pneumoniae* and *E. cloacae*, respectively. The same trend was observed using glucose (15 g/L) as the standard substrate.

**Table 2:** Biohydrogen production (mH_2_/L) from *K. pneumoniae, E. cloacae* and coculture using thermochemical hydrolysate of *O. acuminata*

Cultures	*K. pneumoniae*		*E. cloacae*		Coculture	
Time (h)	Glucose	*O. acuminata*	Glucose	*O. acuminata*	Glucose	*O. acuminata*
12	372 ± 9.62	350 ± 9.62	238 ± 9.62	216 ± 9.62	500 ± 20.45	400 ± 18.56
24	578 ± 25.45	498 ± 25.45	422 ± 16.66	344 ± 16.66	960 ± 30.25	890 ± 25.12
36	655 ± 9.62	583 ± 16.66	486 ± 16.66	433 ± 16.66	1160 ± 32.89	1250 ± 19.34
48	927 ± 9.62	855 ± 9.62	612 ± 9.62	572 ± 25.45	1300 ± 20.56	1520 ± 33.5
60	983 ± 16.66	933 ± 16.66	684 ± 16.66	683 ± 25.45	–	–
72	1046 ± 16.66	1022 ± 9.62	835 ± 25.45	813 ± 9.62	–	–
F-value	1254.45*	22145.98*	1365.98*	653.23*	2653.2*	1563.4*

Values represent means of three replicates ± standard error; * indicate significant differences (p ≤ 0.05)

Moreover, the fermentation process was completed within 72 h in monoculture with significant differences at different incubation periods (*K. pneumoniae: F* = 22,145.98, df = 5, *p* < 0.001*; E. cloacae: F* = 653.23, df = 5,* p* < 0.001*)*, while the coculture fermentation was achieved in only 48 h with a significant difference at different fermentation periods (*F* = 1563.4, df = 3, *p* < 0.01) and generated a superior hydrogen evolution efficiency, hydrogen production, and rate hydrogen yield. Analogous results were obtained by Laxman Pachapur et al., who stated that coculture enhances H_2_ production compared to a single bacterial culture [61]. Coculture offers synergistic effects with higher H_2_ yield than monoculture [62], requiring less fermentation period and supplying more H_2_ [63, 64]. The coculture system works in tandem, guaranteeing process stability and improved performance than monocultures, as well as having overcome the limitations of axenic cultures with economic and methodological advantages over enzymatically hydrolyzed cellulose by eliminating the use of reductants during H2 production [65–67]. In addition, the coculture system decreases the incubation time from 3 days to almost 2 days, allowing for additional runs throughout the year and lowering the total cost of H2 generation. In coculture systems, predetermined microbial strains execute complicated activities such as substrate hydrolysis, metabolism exchange, simultaneous substrate consumption and working on several substrates while lowering the working time and releasing greater hydrogen than in monoculture systems [68]. However, chemical pretreatment was insufficient to release all reducing sugars in *O. acuminata* biomass and may generate compounds that inhibit biohydrogen fermentation. Consequently, biological and biological treatment + Mg-Zn Fe_2_O_4_ NPs were used to enhance hydrogen production using the coculture of *K. pneumoniae* and *E. cloacae*.

#### Biological and biological pretreatment + Mg-Zn Fe_2_O_4_ NPs treatment

Biological pretreatment of microalgal biomass was used to augment their fermentable contents for hydrogen-producing bacteria. As a result, biohydrogen production from mixed cultures was increased to 2645 mLH_2_/L in *O. acuminata* at 48 h with significant differences among fermentation periods (F = 1632.6, df = 3, p < 0.01) (Table 3) representing 74% more than chemical pretreatment. This may be because enzymes of biological pretreatments contain amino acids rich in nitrogen that maintains proper nutrient levels throughout fermentation, enhance the growth and protect fermentative microorganisms from osmotic stress [69].

**Table 3:** Biohydrogen production (mLH_2_/L) from* coculture* of* K. pneumoniae* and *E. cloacae* using hydrolysate of *O. acuminata* by biological treatment

Incubation period (h)	Glucose	Biological treatment	Biological treatment + Mg-Zn Fe_2_O_4_ NPs
12	500 ± 20.45	500 ± 22.65	590 ± 36.45
24	960 ± 30.25	1765 ± 23.25	2214 ± 70.21
36	1160 ± 32.89	2134 ± 37.25	2954 ± 66.3
48	1300 ± 20.56	2645 ± 31.02	3254 ± 36.54
F-value	2653.2*	1632.6*	9561*

Values represent means of three replicates ± standard error; * indicate significant differences (p ≤ 0.05)

**Fig. 10:**
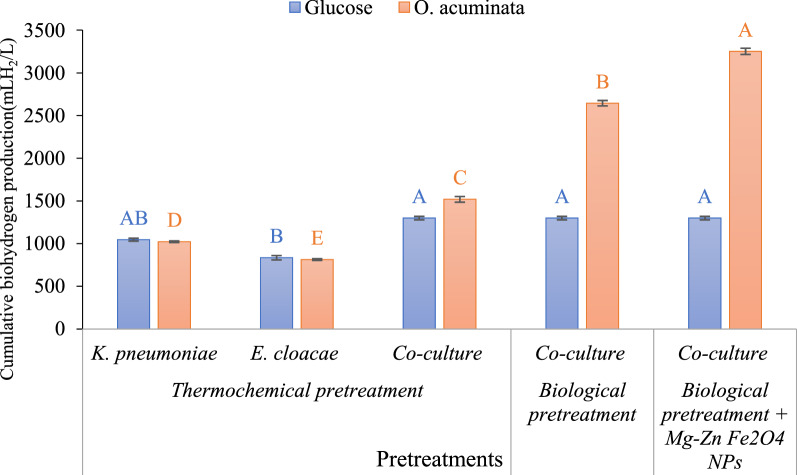
Cumulative biohydrogen production after 48 h from *K. pneumoniae, E. cloacae* and their coculture using different hydrolysates of *O. acuminata* compared to glucose standard*.* Each value represents the mean value of three independent replicates. Different capital letters in each plotted series indicate significant differences at p ≤ 0.05 using Duncan's test

Biological treatment + Mg-Zn Fe_2_O_4_ NPs significantly induced hydrogen production rates compared to the other pretreatments. Accumulative hydrogen production of the coculture was enhanced to attain 3254 mLH_2_/L at 48 h with substantial differences amongst fermentation periods (F = 9561, df = 3, p < 0.001) (Table 3). These amounts represent 1.14-fold when compared with chemical treatment, as shown in Fig. 10. This yield was the highest amount developed among all applied treatments. Abdel-Kader et al. observed that hydrogen production of the mixed cultures of bacterial strain (*Rhodobacter* sp, *Rhodopseudomonas palustris*) was improved to attain 4700 mLH_2_/ L culture [14]. Srivastava et al. recorded an increase in hydrogen yield to 1820 mLH_2_/L via *Bacillus subtilis* PF_1 by using NiFe_2_O_4-_NPs treated cyanobacteria biomass [18]. Moreover, the remaining nanoparticles in the biological pretreatment hydrolysate may have a crucial role in enhancing the hydrogen accumulation. The iron and its oxide NPs clearly activate the main enzymes of hydrogen generation and boost their activities by supplying iron for synthesis and maintaining the essential enzymes' molecular structure [70–72]. Moreover, the use of nanoparticles in stabilizing enzymes has been shown to increase sugar production and subsequently, bio-hydrogen production in a shorter time, thus speeding up the fermentation process [73].

### Hydrogen evolution efficiency

Hydrogen evolution efficiency (HE) was estimated as attributed to reducing sugar (RS) (consumed sugar) as mL H_2_/g RS and hydrogen yield (mL H_2_/g DW) as shown in Table 4. The maximum HE value)216.9 mL H_2_/g RS,( hydrogen yield (92.9 mL H_2_/g Dw, 4.15 mmol/g DW) and hydrogen production rate (67.7 mL/L/h) was obtained from biological treatment + Mg-Zn Fe_2_O_4_ NPs of *O. acuminata* using co-cultures bacterial culture with an increase of 1.62, 1.14 and 1.13-fold, respectively compared to thermochemical pretreatment of coculture. These findings are in close agreement with that presented by Wieczorek et al*.*, who observed that hydrogen yield differs significantly in the range of 11–135 mL H_2_/g from pretreated biological microalgae [74]. Wang and Yin, indicated that hydrogen yield by anaerobic sludge from microalgae pretreated by the combined methods varies in the range of 33.5–92.68 mL H_2_/g [75].

**Table 4:** Hydrogen evolution efficiency (mLH_2_/g RS), hydrogen yield (mL/g DW) and hydrogen rate (mL/L/h) from* K. pneumoniae* and *E. cloacae* and their mixed coculture using hydrolysates* O. acuminata* of by different treatments

Cultures	*K. pneumoniae*		*E. cloacae*		coculture			
Properties	Glucose (15 g/L)	*Thermochemical pretreated O. acuminata* (35 g/L)	Glucose (15 g/L)	*Thermochemical pretreated O. acuminata* (35 g/L)	Glucose (15 g/L)	*Thermochemical pretreated O. acuminata* (35 g/L)	*Biological pretreated O. acuminata* (35 g/L)	*Biological treated* + *NPs O. acuminata* (35 g/L)
Initial sugar (g/L)	15 ± 0.47	16 ± 0.57	15 ± 0.47	16 ± 0.57	15 ± 0.47	16 ± 0.57	31 ± 0.54	45 ± 1.36
Residual sugar (g/L)	3.1 ± 0.17	5.2 ± 0.27	3.2 ± 0.12	5.2 ± 0.65	2.6 ± 0.12	4.6 ± 0.21	17 ± 0.63	29.34 ± 1.07
Consumed sugar (g/L)	11.9 ± 0.13	10.8 ± 0.63	11.8 ± 0.39	10.8 ± 0.64	12.4 ± 0.8	11.4 ± 0.76	14 ± 0.52	15.66 ± 0.9
Total hydrogen (mL H_2_/g RS)	87.9 ± 2.3	94.6 ± 3.28	70.8 ± 1.6	75.3 ± 2.5	104.8 ± 3.6	133.3 ± 4.2	188.9 ± 5.3	216.9 ± 4.69
Hydrogen yield (mL H_2_/g DW)	69.8 ± 1.2	29.2 ± 0.8	55.7 ± 2.3	23.2 ± 1.7	86.7 ± 2.2	43.4 ± 1.6	75.6 ± 1.6	92.9 ± 1.68
hydrogen rate (mLH_2_/L/h)	14.52 ± 0.6	14.19 ± 0.91	11.5 ± 0.62	11.2 ± 0.25	27.08 ± 1.03	31.66 ± 2.01	55.10 ± 2.1	67.7 ± 3.4

Values represent means of three replicates ± standard error

## Conclusions

Based on the current results, it can be concluded that biological pretreatment maximizes reducing sugar release, avoids generating fermentation-inhibiting compounds and reduce chemical inputs compared to thermochemical pretreatments. Mg-Zn Fe2O4-NPs enhanced Trichoderma harzianum cellulolytic activity to give the bacteria with the largest easily fermentable sugar (230.48 mg/g) to produce the highest H_2_ evolution among other pretreatments owing to their high hydrolase content and therefore ability to hydrolyze O. acuminata biomass. Furthermore, the coculture of the two studied bacteria, *K. pneumoni* and E. cloacae, produced more hydrogen (3254 mLH_2_/L), reflecting synergistic consumption of available resources. Therefore, biological pretreatment + Mg-Zn Fe_2_O_4_-NPs and coculture dark fermentation are helpful for biohydrogen production using microalgae biomass.

## Material and methods

### Materials

All reagents and chemicals used in the current study were of analytical grade. They were purchased from the Sinopharm Chemical Reagent Co., Ltd. (Huangpu, Shanghai, China) or Sigma-Aldrich Chemical Co. (St (St. Louis, MO, USA).

Nano ferrite nanoparticles in the chemical formula Mg_1-x_Zn_x_Fe_2_O_4_ (x = 0.1) were kindly provided by Dr. Ahmed Henaish, Physics Department, Faculty of Science, Tanta University, Tanta, Egypt. Mg-Zn Fe_2_O_4_-NPs were synthesized using mechanical ball milling method [76].

### Microalgal species and culture media

*Oscillatoria* sp. isolated from channels and sewage in Gharbia Governorate was used as model microorganisms in the present study. The sub-culturing method was used to isolate and purify axenic cultures to obtain axenic cultures. The cyanophyte was identified morphologically and authenticated according to standard references [77] and AlgaeBase digital resources [78].

### Culture technique for microalgal species

*Oscillatoria* sp. was inoculated (OD = 0.06) into 500 mL Erlenmeyer flasks containing autoclaved 250 mL of the BG11 (Blue-Green algae) medium [79]. The culture was sparkling by air pumps and incubated at 25 ± 2 ºC under continuous daylight fluorescent tubes giving a light intensity of 80 µmol. Photon m^−2^ s^−1^ [80].

### Molecular genetic characterization of microalgal species

*Oscillatoria* sp. was genetically identified based on 16S rRNA. Total genomic DNA was extracted and purified from the isolated species using the GeneJet Genomic DNA purification Mini Kit (Thermo Scientific) following the manufacturer's procedure. A CreaCon thermal cycler was used to do the amplicon (Holand). Green taq (DreamTaq) master mix (Thermo Scientific) was employed as directed by the manufacturer for gene amplification. 16S rRNA primer was applied to amplify cyanobacterial 16S rRNA region using universal forward primer 27F (5′-AGAGTTTGATCCTGGCTCAG-3′) and 1492R (5′- CTACGGCTACCTTGTTACGA- 3′). The thermal cycler conditions were initiated with denaturation for 3 min at 94 ℃ followed by 35 incubation cycles, each consisting of 1 min denaturation at 94 ℃, 1.5 min annealing at 59 ℃, 2 min elongation at 72 °C and a final 5 min elongation at 72 ℃ [81, 82].

PCR products were purified via Gene JET PCR Purification Kit (Thermo Scientific). ABI PRISM^®^ 3100 Genetic Analyzer was applied for PCR products and operated by Macrogen In. Seal, Korea. To identify the closely related species, the purified products were sequenced and aligned with various 16S rRNA get through the nBLAST search (http://www.ncbi.nlm.nih.gov/blast). Clustal W with the default settings of MEGA 11 software [83] was used to align the sequences. In addition, MEGA 11 software was used to generate a dendrogram based on the neighbor-joining (NJ) technique [84] using the parameter distance (PD) [85].

### Estimation of growth, photosynthetic pigments, total soluble protein and carbohydrate content of microalgal species

The growth curve of each microalgal species was detected by optical density (OD 750 nm) and dry weight day after day for 3 weeks [86]. Protein content was determined according to the Bradford method [87]. Total carbohydrates were estimated by spectrophotometry using phenol–sulphuric acid against glucose standard [88] according the procedures of the previous studies [81, 89].

### Microalgal biomass pretreatments

Microalgal biomass was pretreatment by thermochemical, biological, and biological + Mg-Zn Fe_2_O_4_-NPs before being used as a substrate for fermentation.

#### Thermochemical pretreatment

In this pretreatment, different concentrations of H_2_SO_4_ (0.5%, 1%, 1.5%, 2%) with a ratio of 10 ml solution/1 gm microalgal biomass were used [90]. This pretreatment saccharified the complex sugar in the algal biomass to simple. Entire hydrolysates were neutralized with 1 M NaOH to be used in dark fermentation.

#### Biological treatment

*Trichoderma harzianum* Ps-2 of high cellulolytic activity, according to our previous study [39] was used as the source of hydrolytic enzymes. First, it was identified using the ITS region and submitted in the gene bank (Accession no: OP264067) (Additional file 1: Figure S1). Then, two successive steps were performed to improve the cellulolytic activity of this fungi. Firstly, different substrates of agricultural wastes (wheat straw, rice straw, and wheat bran) were used in solid-state fermentation (SSF). Then, five grams of each dry, milled (0.5–1 cm) substrate were mixed with 2.5 g yeast extract/L deionised water, with the moisture level maintained at 60%. Next, a fungal spore suspension (10^6^ cells/mL) was aseptically inoculated in flasks (5%; w/v of the substrate) and incubated at 30 °C for 5 days [91].

Secondly, different concentrations of Mg-Zn Fe_2_O_4_-NPs (20 to 80 ppm) were applied for the suitable agriculture waste substrate in SSF under the same condition. The crude enzymes were extracted by adding Na_3_C_6_H_5_O_7_ buffer (50 mL, 0.5 M, pH 4.8), which contained 0.1% Tween 80 and incubated in a shaker for 1 h at 160 rpm. The entire contents were centrifuged at 6000 rpm at 4 °C for 20 min. The supernatant of the crude enzymes was analyzed for cellulolytic enzymes. The optimum substrate was set for the following experiments.

### Effect of enzyme dose and incubation duration of cyanobacterial biomass

The crude enzyme mix was homogenized with the cyanobacterial suspension (0.3 g in 10 mL) in two ratios (1:1 and 1:2; crude enzyme: algal suspension) to make the final concentration of the crude enzyme 50% and 33% (v/v). The efficacy of the enzymatic activity was estimated after 1 h and 24 h of incubation.

### Enzyme action on cyanobacterial biomass

The enzyme action was studied by estimating the reducing sugars, Fourier Transform Infrared Spectroscopy (FTIR), and scanning electron microscope (SEM) analysis to visualize the morphologic changes in the biomass texture**.**

#### Fourier Transform Infrared Spectroscopy (FTIR)

The FT-IR spectrum analysis was conducted using a Vertex 80v powder FT-IR spectrometer (FTIR Bruker) (model, TENSOR27). Two mg of dried *Oscillatoria* biomass (treated and untreated) samples were mixed well with 200 mg of potassium bromide to make pressed discs for FT-IR measurement in the frequency range of 500–4000 cm^−1^ [92].

#### Scanning electron microscopic (SEM) analysis

SEM analysis was used to analyze the morphological and micro-structural variations of the treated and untreated samples using SEM (Tescan vega 3 SBU, Czech Republic) at an accelerating voltage of 220 keV. The samples were displayed on aluminum microscopy stubs using carbon tape, then covered with a thin layer of gold (Au) for 120 s using Quorum technique Ltd, sputter coater (Q150t, England) [93].

### Biohydrogen production

#### Isolation and identification of biohydrogen-producing bacteria

The bacterial strains were isolated from Tanta, Egypt’s wastewater treatment plant (Elmorashaha). The sludge was blended with sterile distilled water before being dispersed onto sterilized nutrient agar plates and incubated at 37 °C in a N2-flushed anaerobic incubator. The isolated colonies were then inoculated in a cuvette with a Durham tube and cultivated in an anaerobic incubator. The Durham tube would detect evolved gas instantly. The syringe and gas chromatography were used to quantify the volume and composite of evolved gas within the syringe, and the strains with the greatest hydrogen generation yield or rate were chosen [94]. The bacterial isolates were morphologically and biochemically characterized according to Bergey's manual of systematic bacteriology [95]. Also, these isolated bacteria were further identified by 16S rRNA sequencing according to "Influence of different pretreatment methods on biomass hydrolysis" Section.

### Experimental design of biohydrogen production

Biohydrogen production was carried out via dark batch fermentation of hydrolysate of the three pretreatments of microalgal biomass (thermochemical, biological, and biological + Mg-Zn Fe_2_O_4_-NPs) using the two isolated bacteria individually or their coculture. Batch experiments were performed in 100 mL bottles containing the autoclaved anaerobic medium with 10% V/V of pre-enriched pure bacterial culture. The bottles were sealed with sterilized air-tight rubber caps and parafilm, then purged with nitrogen gas for 3 min to drop the dissolved oxygen concentration down to zero. The bottles capped with rubber stoppers were stirred using the magnetic stirrer plate to help evolve hydrogen [14]. The heater and temperature probe of the magnetic stirrer held the water temperature constant at 37 ℃. The gas was trapped in a water inverted cylinder and connected with 2 M NaOH solution to absorb CO_2_ produced during fermentation**.** Hydrogen yield was measured by the liquid displacement technique.

### Determination of the produced biohydrogen by gas chromatography (GC)

The evolved biohydrogen gas was determined by gas chromatograph apparatus (Thermo Scientific TRACE GC Ultra) equipped with a thermal conductivity detector (TCD) and Shin Carbon packed column (ST 80/100 2 m, 2 mm ID). Argon was used as carrier gas. For each pretreatment, triplicates were performed, and the average values were taken as the final result. A control test was performed using a fermentative medium containing glucose. An additional blank assay with only an anaerobic medium without any substrate or inoculum also acted as a negative control [96].

## Supplementary Information

**Additional file 1: Figure S1.** Neighbor-Joining (NJ) dendrograms showing the isolated Trichoderma harzianum based on 18S rRNA nucleotide sequences, respectively. Bootstrap values higher than 70 are shown below the branches of the trees.

## Data Availability

This article has all the data that was created or assessed during this study.
