# Supplementary material for: Application of a novel biological-nanoparticle pretreatment to Oscillatoria acuminata biomass and coculture dark fermentation for improving hydrogen production
Source: Microb Cell Fact. 2023 Feb 22;22:34. doi: 10.1186/s12934-023-02036-y (PMC9948338; doi:10.1186/s12934-023-02036-y)
Supplement: Supplementary file 1 — Additional file 1: Figure S1. Neighbor-Joining (NJ) dendrograms showing the isolated Trichoderma harzianum based on 18S rRNA nucleotide sequences, respectively. Bootstrap values higher than 70 are shown below the branches of the trees. [file 12934_2023_2036_MOESM1_ESM.docx]

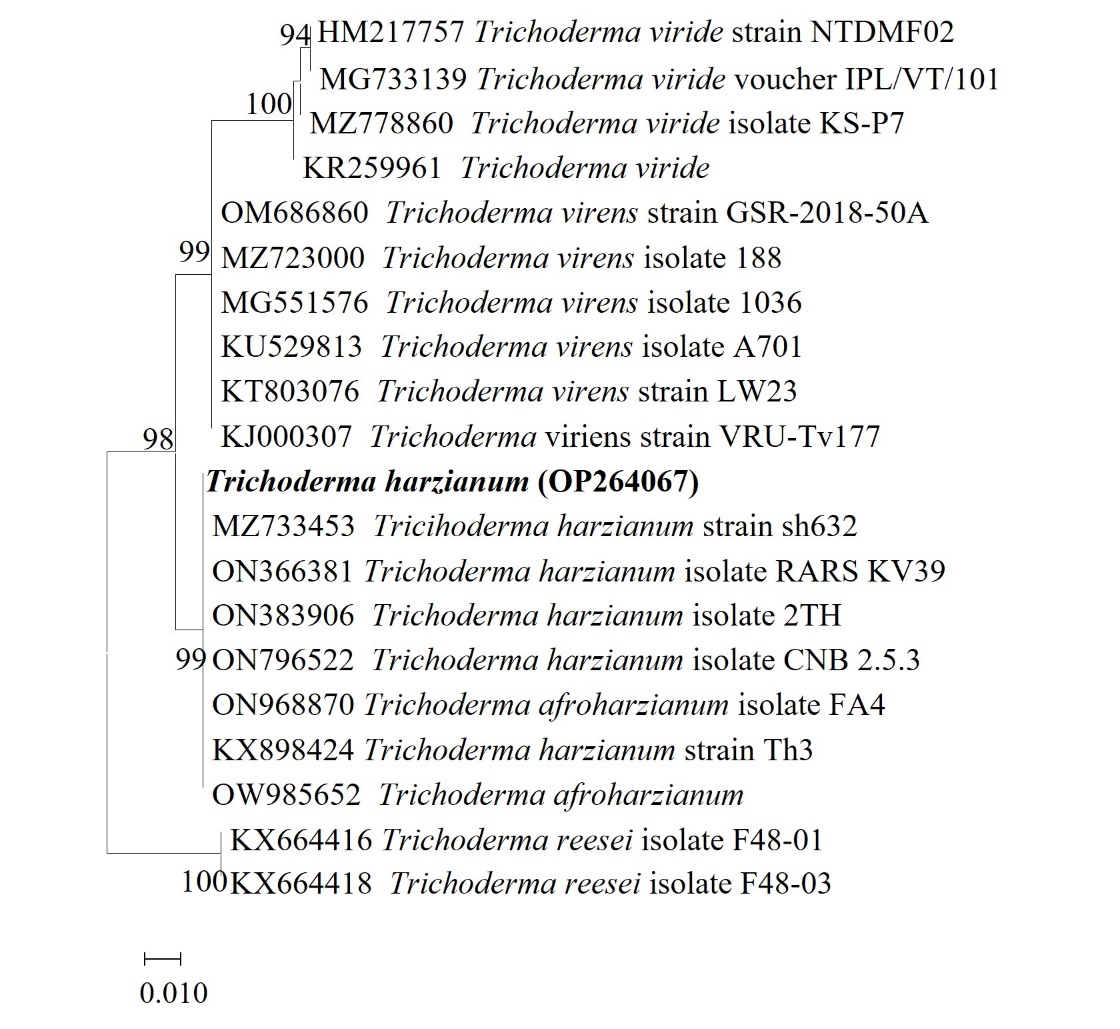


Fig.S1. Neighbor-Joining (NJ) dendrograms showing the isolated *Trichoderma harzianum* based on 18S rRNA nucleotide sequences, respectively. Bootstrap values higher than 70 are shown below the branches of the trees.
